# Performance of the CKD-EPI and MDRD equations for estimating glomerular filtration rate: a systematic review of Latin American studies

**DOI:** 10.1590/1516-3180.2020.0707.R1.150321

**Published:** 2021-08-09

**Authors:** Ana Brañez-Condorena, Sergio Goicochea-Lugo, Jessica Hanae Zafra-Tanaka, Naysha Becerra-Chauca, Virgilio Efrain Failoc-Rojas, Percy Herrera-Añazco, Alvaro Taype-Rondan

**Affiliations:** I Undergraduate Student, Facultad de Medicina and Asociación para el Desarrollo de la Investigación Estudiantil en Ciencias de la Salud, Universidad Nacional Mayor de San Marcos, Lima, Peru.; II MD. Methodologist, EsSalud, Instituto de Evaluación de Tecnologías en Salud e Investigación, Lima, Peru.; III MD, MSc. Professor, Escuela de Medicina, Universidad Científica del Sur, Lima, Peru.; IV Midwife. Methodologist, EsSalud, Instituto de Evaluación de Tecnologías en Salud e Investigación, Lima, Peru.; V MD, MSc. Methodologist, EsSalud, Instituto de Evaluación de Tecnologías en Salud e Investigación, Lima, Peru; and Researcher, Unidad de Investigación para la Generación y Síntesis de Evidencias en Salud, Universidad San Ignacio de Loyola, Lima, Peru.; VI MD, MHEd. Researcher, Universidad Privada San Juan Bautista, Lima, Peru; and Assistant Manager, EsSalud, Instituto de Evaluación de Tecnologías en Salud e Investigación, Lima, Peru.; VII MD, MSc. Methodologist, EsSalud, Instituto de Evaluación de Tecnologías en Salud e Investigación, Lima, Peru; and Researcher, Unidad de Investigación para la Generación y Síntesis de Evidencias en Salud, Universidad San Ignacio de Loyola, Lima, Peru.

**Keywords:** Renal insufficiency, chronic, Glomerular filtration rate, Latin America, Systematic review [publication type], Meta-analysis [publication type], Chronic renal failure, Chronic kidney disease, Diagnoses, Screening

## Abstract

**BACKGROUND:**

The most-used equations for estimating the glomerular filtration rate (GFR) are the CKD Epidemiology Collaboration (CKD-EPI) and Modification of Diet in Renal Disease (MDRD) equations. However, it is unclear which of these shows better performance in Latin America.

**OBJECTIVE:**

To assess the performance of two equations for estimated GFR (eGFR) in Latin American countries.

**DESIGN AND SETTING:**

Systematic review and meta-analysis in Latin American countries.

**METHODS:**

We searched in three databases to identify studies that reported eGFR using both equations and compared them with measured GFR (mGFR) using exogenous filtration markers, among adults in Latin American countries. We performed meta-analyses on P30, bias (using mean difference [MD] and 95% confidence intervals [95% CI]), sensitivity and specificity; and evaluated the certainty of evidence using the GRADE methodology.

**RESULTS:**

We included 12 papers, and meta-analyzed six (five from Brazil and one from Mexico). Meta-analyses that compared CKD-EPI using creatinine measured with calibration traceable to isotope dilution mass spectrometry (CKD-EPI-Cr IDMS) and using MDRD-4 IDMS did not show differences in bias (MD: 0.55 ml/min/1.73m2; 95% CI: -3.34 to 4.43), P30 (MD: 4%; 95% CI: -2% to 11%), sensitivity (76% and 75%) and specificity (91% and 89%), with very low certainty of evidence for bias and P30, and low certainty of evidence for sensitivity and specificity.

**CONCLUSION:**

We found that the performances of CKD-EPI-Cr IDMS and MDRD-4 IDMS did not differ significantly. However, since most of the meta-analyzed studies were from Brazil, the results cannot be extrapolated to other Latin American countries.

**REGISTRATION:**

PROSPERO (CRD42019123434) - https://www.crd.york.ac.uk/prospero/display_record.php?ID=CRD42019123434.

## INTRODUCTION

Chronic kidney disease (CKD) is a public health problem: in 2014, 10.6% of adults aged over 30 years had stage 3-5 CKD.^1^ In 2017, CKD caused 35,800,000 disability-adjusted life-years (1.4% of all disability-adjusted life-years) worldwide,^2^ and 1,230,200 deaths (2.2% of all deaths).^3^

Assessing the glomerular filtration rate (GFR) is the cornerstone for performing adequate screening, diagnosis and classification of CKD.^4^ However, the methods used for directly measuring GFR (measured GFR, mGFR) require use of exogenous filtration markers and are laborious and costly. Thus, some equations are routinely used to obtain estimated GFR (eGFR) from endogenous markers such as creatinine^5^ or serum cystatin C.^6^ The most commonly used equations are the CKD Epidemiology Collaboration (CKD-EPI) and the Modification of Diet in Renal Disease (MDRD) equations.^7^

The MDRD equation originally used six variables (MDRD-6): serum creatinine, urea, albumin, age, sex and ethnicity.^8^ A later version used only four variables (MDRD-4), excluding serum urea and albumin.^9^ Most recently, the MDRD-4 was re-edited to use creatinine measured with calibration traceable to isotope dilution mass spectrometry (IDMS).^10^^,^^11^

The CKD-EPI originally used the same four variables of the MDRD-4.^12^ Later, other CKD-EPI equations were developed, which used serum cystatin C instead of creatinine,^13^ or used both serum creatinine and cystatin C.^14^

Differences in the performance of these equations across certain ethnic groups have been reported,^15^^-^^18^ and attributed to differences in the production and excretion of creatinine.^19^ This, in turn, is related to diet (protein intake) and muscle mass (endogenous production of creatinine), which vary according to ethnicity.^19^^-^^21^ Thus, it is possible that results from regions with different ethnic compositions such as Europe or North America, which are mostly Caucasian and secondly, Blacks and Hispanics, cannot be extrapolated to Latin American populations that are composed of a mixture of Amerindians, Mestizos, Blacks, Asians and Caucasians.^22^

## OBJECTIVE

Latin American stakeholders and practitioners need to know which equation has the best diagnostic performance in their specific context, in order to better inform their decisions. Therefore, we conducted a systematic review with the aim of comparing the performance of the CKD-EPI and MDRD equations for estimating the GFR in Latin American countries, and we evaluated the certainty of the evidence using the Grading of Recommendations Assessment, Development and Evaluation (GRADE) approach.

## METHODS

The study protocol was registered in PROSPERO (CRD42019123434). We performed a systematic review following the Preferred Reporting Items for Systematic Reviews and Meta-Analysis (PRISMA) guidelines.^23^

### Literature search and study selection

In this systematic review, we included original observational studies that were performed in Latin American countries and compared both the CKD-EPI and the MDRD equation with mGFR (the gold standard, measured using any exogenous filtration markers such as inulin, iohexol, iothalamate, 51Cr-EDTA or DTPA, among others) in adult populations (≥ 18 years). We did not exclude any study on the basis of language or any other criteria.

We performed a two-step sensitive search. First, we carried out a literature search in PubMed and Scopus in January 2019, and in “Biblioteca Regional de Medicina” (BIREME) in February 2019. The search strategy for each database or virtual library is shown in **Supplementary Material 1** (for all supplementary material, see https://doi.org/10.6084/m9.figshare.14614788.v1).

Duplicated records were removed using the EndNote software. Later, two researchers (ABC and NBC) independently selected abstracts for full-text review and final inclusion. Any differences were resolved by a third researcher (JHZT).

Secondly, we searched the lists of references of all studies included, and the lists of articles that cited each of the studies included (through Google Scholar), in order to identify other studies that fulfilled the inclusion criteria.

### Data extraction

Two researchers (ABC and NBC) independently extracted data from each article that met the inclusion criteria, using a standardized Microsoft Excel sheet. Any differences were resolved by a third researcher (JHZT).

The following variables were extracted from each study: first author, year of publication, country, design (prospective or retrospective), population characteristics (inclusion and exclusion criteria, number of participants, sex, age, ethnic group, CKD diagnosis and CKD etiology), intervention (type of MDRD and CKD-EPI equations), gold standard (exogenous filtration marker), mGFR, eGFR and numerical results from diagnostic measurements.

The main diagnostic measurement comprised bias (defined as the mean of the difference between eGFR and mGFR), P30 (percentage of results of eGFR that did not deviate more than 30% from mGFR) and accuracy measurements (sensitivity, specificity and area under the curve).

Other measurements made included the following: precision (defined as one standard deviation of bias, or as the interquartile range), bias% (mean of the difference between eGFR and mGFR, as a function of mGFR), P15, P10, combined root mean square error (CRMSE), Pearson coefficient, intraclass correlation coefficient, kappa coefficient and limits of agreement (defined as bias ± 2 standard deviations).

When there were doubts about some information reported in the studies, we sent an email to the authors in order to clarify the information. 

### Risk of bias and certainty of evidence

Two researchers (NBC and VEFR) assessed the four risk-of-bias domains of the Quality Assessment of Diagnostic Accuracy Studies (QUADAS-2) tool:^24^ patient selection, index test, reference standard and flow and timing. In any cases of disagreement, a consensus was achieved together with a third researcher (JHZT).

We used the GRADE methodology^25^ to report our certainty regarding the evidence of accuracy of the diagnostic test results. To show this certainty, we created tables of summary of findings (SoF), in accordance with the GRADE specifications.^26^^,^^27^

### Statistical analyses

When possible, we performed meta-analyses on P30, bias, sensitivity and specificity. This was done when studies compared similar equations, showed their confidence intervals or standard deviations, or enabled calculation of these values.

For P30 and bias, we calculated mean differences (MD) and their 95% confidence intervals (95% CI). For sensitivity and specificity, we built a 2 x 2 table when possible. As there were fewer than four studies to meta-analyze, we could not perform a meta-analytical hierarchical regression for diagnostic accuracy. Instead, we performed a meta-analysis of proportions using the exact binomial distribution. We assessed heterogeneity using an I^2^ statistic and used random-effects models when I^2^ was higher than 40%. 

For bias and P30, we performed a subgroup analysis according to the presence of CKD (using the cutoff of 60 ml/min/1.73 m^2^), since a previous systematic review showed that the eGFR equation performance varies across these subgroups^28^. We could not perform a subgroup analysis for comorbidities since no more than one study assessed the same version of the equation in any of the comorbidity groups. The data were processed using the Review Manager (RevMan) [Computer program], version 5.4.1 (Copenhagen: The Nordic Cochrane Centre, The Cochrane Collaboration, 2020).

### Ethics committee approval

This was not applicable since this review did not directly involve human participants.

## RESULTS

### Studies characteristics

**Figure 1:**
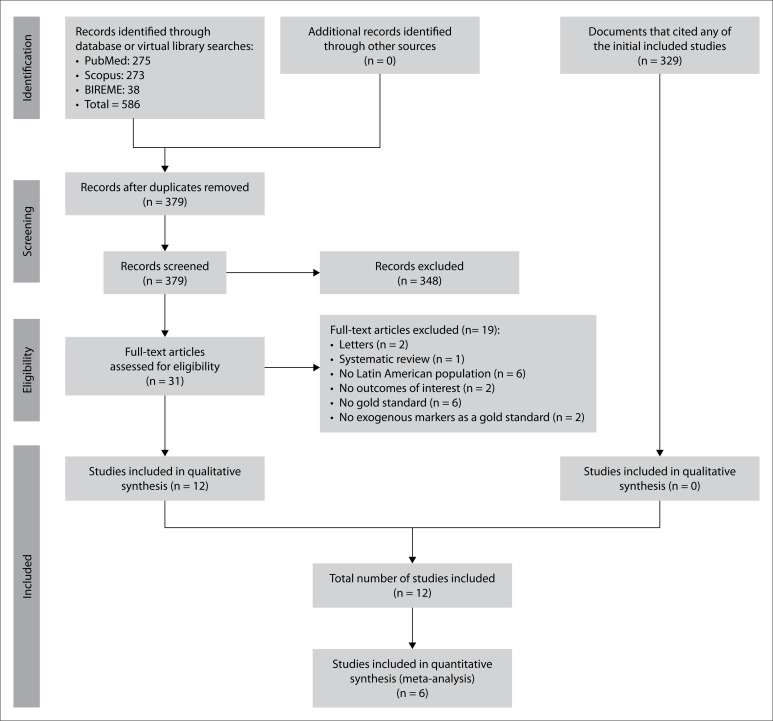
Flow diagram summarizing the process of searching the literature and selecting studies.

In total, we identified 379 records after removing duplicates. Among these, 31 were considered potentially eligible and we did full-text reviews on them. Nineteen were excluded through this process (reasons are detailed in **Supplementary Material 2**, https://doi.org/10.6084/m9.figshare.14614788.v1) and 12 were included for analysis.^29^^-^^40^ In addition, we did not identify any new studies after searching the lists of references of all the studies included and the lists of articles that cited each of the included studies (done through Google Scholar) (**Figure 1**).

The characteristics of the 12 studies included are summarized in **Table 1** and detailed in **Supplementary Material 3** (https://doi.org/10.6084/m9.figshare.14614788.v1). The numbers of participants ranged from 14 to 354 in these studies. Two studies reported results from the same cohort.^30^^,^^40^ One study^38^ added data from two cohorts, one of which^36^ was also included in our review and the other had not been published as a separate original paper.

**Table 1:** Characteristics of the studies included

Studies that were included in the meta-analyses										
Authors	Country	Population/setting	n	% of females	Age (mean in years)	CKD-EPI	MDRD	Diagnostic measurements (P30, bias, sensitivity, or specificity)^*^	Gold standard	mGFR (mean in ml/min/1.73 m^2^)
Arreola-Guerra et al.^29^	Mexico	Healthy/ hospital	97	41.2	35.8	CKD-EPI-Cr IDMS	MDRD-4 IDMS	**Bias**, P30	^99m^Tc DTPA	102.7
Camargo et al.^31^	Brazil	Healthy/ hospital	55	47	56	CKD-EPI-Cr IDMS	MDRD-4 IDMS	**Bias**, P30	^51^Cr-EDTA	98
		Type 2 diabetics/ hospital	56	56	59	CKD-EPI-Cr IDMS	MDRD-4 IDMS	**Bias**, P30		106
David-Neto et al.^32^	Brazil	Elderly/ renal-transplanted	70	40	65	CKD-EPI-Cr IDMS	MDRD-4 IDMS	**Bias**, P30	^51^Cr-EDTA	47
Lopes et al.^33^	Brazil	Elderly/ community	95	70	85.3	CKD-EPI-Cr IDMS, CKD-EPI cystatin C	MDRD-4 IDMS	**Bias, P30, SE, SP**	Iohexol	55
Silveiro et al.^36^	Brazil	Type 2 diabetics/ hospital	105	50	57	CKD-EPI-Cr IDMS	MDRD-4 IDMS	Bias, **P30**	^51^Cr-EDTA	103
Veronese et al.^38^	Brazil	Healthy, type 2 diabetics, CKD/ community, hospital	354	55	53	CKD-EPI-Cr IDMS	MDRD-4 IDMS	**Bias, P30, SE, SP**	^51^Cr-EDTA	87
**Studies that were not included in the meta-analyses**										
Asnani et al.^30^	Jamaica	Homozygous sickle cell disease/ hospital	98	56	34	CKD-EPI-Cr	MDRD-4	Bias, P30	^99m^Tc DTPA	94.91
Asnani et al.^40^	Jamaica	Homozygous sickle cell disease/ hospital	98	56	34	CKD-EPI-Cr, CKD-EPI-cystatin C	MDRD-4	Bias, P30	^99m^Tc DTPA	94.9
Lujan et al.^34^	Argentina	Healthy/ potential donor	85	54	41	CKD-EPI-Cr IDMS	MDRD-4 IDMS	Bias, SE, SP	Non-radiolabeled iothalamate	116
Martinez-Martinez et al.^35^	Mexico	SLE/ hospital	14	100	32.5	CKD-EPI-Cr IDMS	MDRD-4 IDMS	Bias, P30	Non-radiolabeled iothalamate	Not mentioned
Trimarchi et al.^37^	Argentina	CKD, healthy/ hospital	300	42	Median: 48.6	CKD-EPI-Cr	MDRD-4		^99m^Tc DTPA	For different stages of CKD: Control: 81.53 1: 95.26 2: 70.05 3: 45.59 4: 22.60 5: 11.18
Zanocco et al.^39^	Brazil	CKD, healthy/ hospital	244	57	Males: 40.6; females: 42.6	CKD-EPI-Cr IDMS	MDRD-4 IDMS	Sensitivity and specificity	Iohexol	61.31

CKD = chronic kidney disease; SLE = systemic lupus erythematosus, SE = sensitivity, SP = specificity; mGFR: measured glomerular filtration rate. Asnani et al.^30^ and Asnani et al.^40^ evaluated the same cohort; Veronese et al.^38^ added data from two cohorts, one of which was Silveiro et al.^36^

^*^In bold: diagnostic measurements included in the meta-analyses.

Regarding the country, six studies were conducted in Brazil,^31^^-^^33^^,^^36^^,^^38^^,^^39^ two in Mexico,^29^^,^^35^ two in Argentina^34^^,^^37^ and two reported results from the same cohort conducted in Jamaica.^30^^,^^40^ Regarding the population, six studies were performed among healthy people,^29^^,^^31^^,^^34^^,^^37^^-^^39^ one among candidates for living kidney donation,^34^ three among type 2 diabetics,^31^^,^^36^^,^^38^ two among the elderly,^32^^,^^33^ one among people with systemic lupus erythematosus (SLE),^35^ two from the same cohort on homozygous SS sickle cell disease^30^^,^^40^ and three among people diagnosed with CKD.^37^^-^^39^

Nine studies compared MDRD-4 using IDMS (MDRD-4 IDMS) and CKD-EPI-Cr using IDMS (CKD-EPI-Cr IDMS),^29^^,^^31^^-^^36^^,^^38^^,^^39^ one compared MDRD-4 IDMS and CKD-EPI cystatin C,^33^ one compared MDRD-4 IDMS and CKD-EPI-Cr-cystatin C,^33^ three compared MDRD-4 without IDMS and CKD-EPI-Cr without IDMS,^30^^,^^37^^,^^40^ one compared MDRD-4 without IDMS and CKD-EPI cystatin C^40^ and one compared MDRD-4 without IDMS and CKD-EPI-Cr-cystatin C.^40^ Out of the nine studies that compared MDRD-4 IDMS and CKD-EPI-Cr IDMS, six could be included in the meta-analyses (five from Brazil and one from Mexico), since the others did not have enough information to estimate standard errors (**Table 1**).

Regarding use of a correction factor for black race, these six studies included this in the MDRD-4 IDMS equation. Five studies (four from Brazil and one from Mexico) used a CKD-EPI-Cr equation that included the correction factor. One study from Brazil^32^ did not included the correction factor in the CKD-EPI-Cr equation: the population of this study (n = 70) was mostly Caucasian (only 12 people aged ≥ 60 years were of other races and the study did not detail which races these were).

### Risk of bias

**Figure 2:**
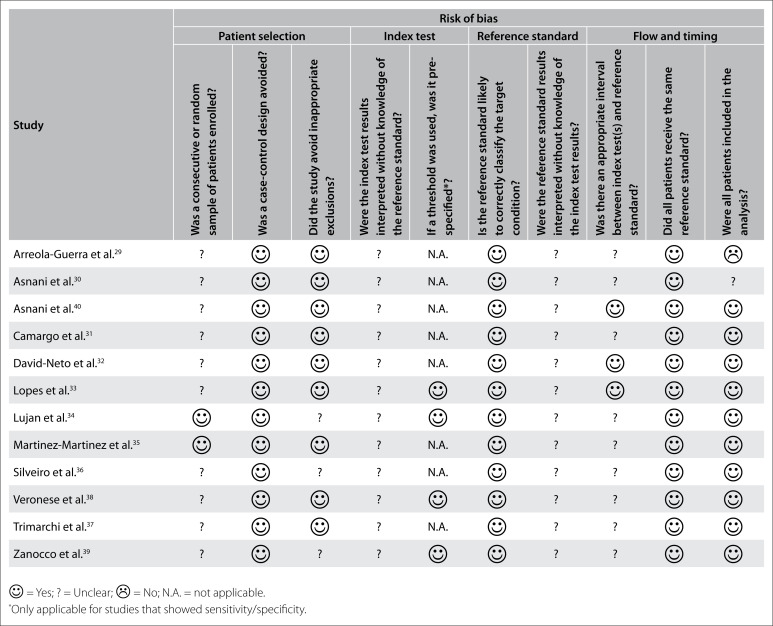
Risk of bias.

Using the QUADAS-2 tool, we found that the risk of bias was uncertain for most studies, regarding patient enrolling, interpretation of index test results without knowledge of the reference standard, interpretation of the reference standard without knowledge of the index test results and the interval between the index and reference standard tests (**Figure 2**).^29^^-^^40^

### Diagnostic outcomes

The results from each study are detailed in **Supplementary Material 4** (https://doi.org/10.6084/m9.figshare.14614788.v1). Meta-analyses could only be performed for the comparison between CKD-EPI-Cr IDMS and MDRD-4 IDMS, since other versions of the equations were not evaluated or were evaluated only in one study for the outcomes of interest.

**Figure 3:**
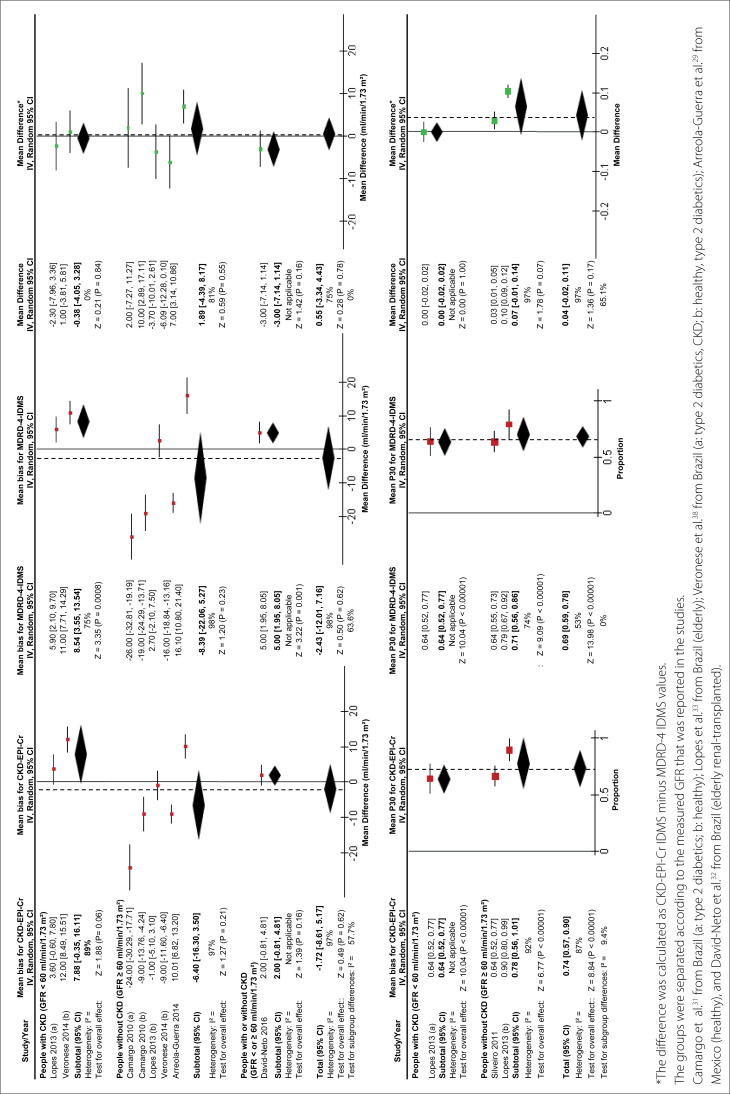
Forest plot for bias and P30.

**Figure 4:**
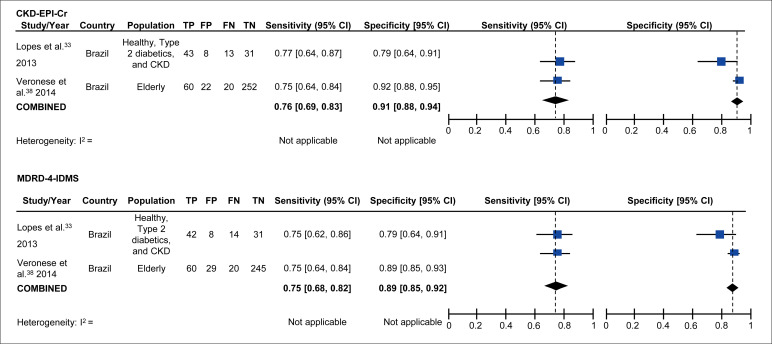
Forest plot for sensitivity and specificity (cutoff of GFR 60 ml/minute/1.73 m^2^).

Meta-analyses on bias and P30 are shown in **Figure 3**. Meta-analyses on sensitivity/specificity (for the cutoff of GFR 60 ml/min/1.73 m^2^) are shown in **Figure 4**.

Regarding bias: meta-analyses on five studies (four performed in Brazil and one in Mexico)^29^^,^^31^^-^^33^^,^^38^ showed no differences between these equations, although point estimates tended to slightly favor the CKD-EPI-Cr IDMS equation (MD: 0.55 ml/min/1.73 m^2^; 95% CI: -3.34 to 4.43). For the record, the CKD-EPI-Cr IDMS advantage is higher (although still not significant) in populations with GFR ≥ 60 ml/min/1.73 m^2^. In addition, these meta-analyses showed that both equations tended to overestimate mGFR in people with CKD and to underestimate it in people without CKD.

Regarding P30: meta-analyses on two studies (both performed in Brazil)^29^^,^^31^^-^^33^^,^^38^ showed a P30 of 74% (95% CI: 57% to 90%) for CKD-EPI-Cr IDMS, and of 69% (95% CI: 59% to 78%) for MDRD-4 IDMS. However, the final mean difference was not compatible with a significant difference, although point estimates tended to slightly favor the CKD-EPI-Cr IDMS equation (MD: 4%; 95% CI: -2% to 11%). It should be noted that the CKD-EPI-Cr IDMS advantage is higher (although still not significant) in populations with GFR ≥ 60 ml/min/1.73 m^2^.

Regarding sensitivity and specificity, two studies (both performed in Brazil)^33^^,^^38^ showed similar sensitivity (76% for CKD-EPI-Cr IDMS and 75% for MDRD-4 IDMS) and specificity (91% for CKD-EPI-Cr IDMS and 89% for MDRD-4 IDMS).

### Certainty of evidence

We used GRADE SoF tables to report the certainty of evidence. Regarding bias and P30, the certainty of evidence was very low for both CKD-EPI-Cr IDMS and MDRD-4 IDMS (**Table 2**). Regarding differences in true positives, true negatives, false positives and false negatives between equations (obtained through sensitivity and specificity), the certainty of evidence was low (**Table 3**).

**Table 2:** Summary of findings of bias and P30

**Question: How good are the performances of the CKD-EPI-Cr IDMS and MDRD-4 IDMS equations for diagnosing CKD in adult populations (≥ 18 years) in Latin America?**			
**Patient or population:** Adults in Latin American countries			
**Settings:** The studies included involved community-dwelling adults and hospital-based patients (mean prevalence of CKD across studies included: 41%)			
**New test:** CKD-EPI-Cr IDMS			
**Comparison test:** MDRD-4 IDMS			
**Reference test:** The measured glomerular filtration rate (mGFR) was taken to be the gold standard and was obtained using the Cr-EDTA single-injection method in four studies, Iohexol clearance in one study, and ^99m^Tc DTPA in one study.			
**Outcome**	**Number of studies (number of participants)**	**Test result (95% CI)**	**Quality of the evidence (GRADE)**
**Bias**			
CKD-EPI-Cr IDMS	5 (727)	**-1.72** (-8.61 to 5.17)	⊕○○○ **VERY LOW**^1^^,^^2^^,^^3^^,^^4^
MDRD4 IDMS		**- 2.43** (-12.01 to 7.16)	⊕○○○ **VERY LOW**^1^^,^^2^^,^^3^^,^^4^
**P30**			
CKD-EPI-Cr IDMS	2 (200)	**73.78%** (58.03 to 89.52)	⊕○○○ **VERY LOW**^3^^,^^5^
MDRD-4 IDMS		**68.83%** (59.21 to 78.44)	⊕○○○ **VERY LOW**^3^^,^^5^

GRADE Working Group grade of evidence. High quality: Further research is very unlikely to change our confidence in the estimate of effect; Moderate quality: Further research is likely to have an important impact on our confidence in the estimates of effect and may change the estimates; Low quality: Further research is very likely to have an important impact on our confidence in the estimates of effect and is likely to change the estimates; Very low quality: We are very uncertain about the estimates. Bias: Defined as the mean of the difference between eGFR (from equations) and mGFR; P30: Defined as the percentage of results for eGFR that did not deviate more than 30% from mGFR. eGFR = estimated glomerular filtration rate; mGFR = measured glomerular filtration rate; CI = confidence interval; CKD = chronic kidney disease; CKD-EPI-Cr IDMS = CKD epidemiology collaboration equation using creatinine with isotope dilution mass spectrometry method to determine creatinine levels; MDRD-4 IDMS = modification of diet in renal disease (with four variables) equation with isotope dilution mass spectrometry method to determine creatinine levels.

^1^It was decided to downgrade the level of evidence due to risk of bias because, in more than 50% of the studies, it was uncertain whether the gold standard and reference results were collected at the same time

^2^It was decided to downgrade the level of evidence due to high heterogeneity between the studies (I^2^ higher than 90%)

^3^It was decided to downgrade the level of evidence due to risk of bias (the gold standard was not the same in all the studies)

^4^It was decided to downgrade the level of evidence due to imprecision (both equations could overestimate or underestimate the real value of the GFR)

^5^It was decided to downgrade by one level due to risk of bias (it was uncertain whether the results for the gold standard and the reference were collected at the same time, and in one of the studies, no analysis was done on the results from some of the participants).

**Table 3:** Summary of sensitivity and specificity findings for the 60 ml/min/1.73 m^2^ cutoff point

**Question: How accurate are the CKD-EPI-Cr IDMS and MDRD-4 IDMS equations for diagnosing CKD in adult populations (≥ 18 years) in Latin America?**					
**Number of participants (Studies)**	449 (2)	**Pooled sensitivity CKD-EPI-Cr IDMS**	**0.76** (95% CI: 0.69 to 0.83)	**Pooled sensitivity MDRD4-IDMS**	**0.75** (95% CI: 0.68 to 0.82)
		**Pooled specificity CKD-EPI-Cr IDMS**	**0.91** (95% CI: 0.88 to 0.94)	**Pooled specificity MDRD4-IDMS**	**0.89** (95% CI: 0.85 to 0.92)
**Test result**		**Number of results per 1,000 patients tested (95% CI)**			
		Baseline risk across studies included: **41%**		**Quality of the evidence (GRADE)**	
		**CKD-EPI-Cr IDMS**	**MDRD4-IDMS**		
**True positives (TP)**		**312** (283 to 340)	**308** (279 to 336)	⊕⊕○○ **LOW** ^1^^,^^2^	
TP absolute difference		**4 more TP in CKD-EPI-Cr IDMS**			
**False negatives (FN)**		**98** (70 to 127)	**102** (74 to 131)		
FN absolute difference		**4 less FN in CKD-EPI-Cr IDMS**			
**True negatives (TN)**		**537** (519 to 555)	**525** (502 to 543)	⊕⊕○○ **LOW** ^1^^,^^2^	
TN absolute difference		**12 more TN in CKD-EPI-Cr IDMS**			
**False positives (FP)**		**53** (35 to 71)	**65** (47 to 88)		
FP absolute difference		**12 less FP in CKD-EPI-Cr IDMS**			

CI = confidence interval; CKD = chronic kidney disease; CKD-EPI-Cr IDMS = CKD Epidemiology Collaboration equation using creatinine with isotope dilution mass spectrometry method to determine creatinine levels; MDRD4 IDMS = Modification of Diet in Renal Disease (with four variables) equation with isotope dilution mass spectrometry method to determine creatinine levels.

^1^It was decided to downgrade the level of evidence due to risk of bias (in both studies, it was uncertain whether a consecutive or random sample of patients was enrolled and whether the results from the index test were interpreted without knowledge of the results of the gold standard)

^2^It was decided to downgrade the level of evidence due to risk of bias (the gold standard was not the same in all the studies).

## DISCUSSION

### Comparison with other studies

We performed meta-analyses on six studies conducted in Latin American countries (five from Brazil, one from Mexico) that compared CKD-EPI-Cr IDMS and MDRD-4 IDMS. No clear differences between these equations were found with regard to bias, P30, sensitivity or specificity. However, point estimates showed a lower bias and a higher P30 (both non-statistically significant) using CKD-EPI-Cr IDMS, in comparison with using MDRD-4.

A previous systematic review among patients in primary care settings searched for studies up to 2017 and included six studies conducted in Latin American countries (all of which were included in our review).^28^ That review found that in studies using IDMS, CKD-EPI-Cr IDMS had lower bias (MD: 2.2 ml/minute/1.73 m^2^; 95% CI: 1.1 to 3.2) and higher P30 (MD: 2.7%; 95% CI: 1.6 to 3.8) than MDRD-4 IDMS. Considering this, it is possible that in our population, as well as in the population reported in the previous review, the CKD-EPI-Cr IDMS equation could really have slightly better performance, which cannot be observed due to the lack of power (given the small sample size and high heterogeneity) and the absence of sufficient data to be considered for inclusion in the meta-analysis on the other studies that evaluated bias and P30.

This presumed advantage of CKD-EPI-Cr IDMS over MDRD-4 IDMS was more evident in studies in which the population did not have CKD (GFR ≥ 60 ml/minute/1.73 m^2^). A similar trend was found in the previous systematic review.^28^ This could be due to the fact that the CKD-EPI-Cr equation was developed in a study in which the mean GFR was higher than the GFR of the study in which the MDRD-4 equation was created (94.5 ml/minute versus 39.8 ml/minute respectively).^12^

### How to better evaluate eGFR in Latin American populations

These equations may not be accurate for all racial groups due to differences in muscle mass and, consequently, differences in creatinine excretion.^21^ Thus, attempts to correct the estimates according to race have been made in these equations using different coefficients for white or black people, but other races have not been taken into account.

Given this limitation, modifications of the formulas have been proposed for several ethnic groups, including Asians,^41^ Japanese,^18^ Chinese,^42^ Pakistanis^43^ and Africans.^15^ However, previous attempts to modify the CKD-EPI-Cr formula for Latin American populations^44^ and a Brazilian population^39^ did not find any significant improvements in the modified formula, compared with the original formula. This may be due to the fact that Latin American populations do not include a single ethnic group, but a confluence of multiple ethnicities from diverse origins, and the profile of each population (in terms of percentage of European-descendant, Afro-descendent or indigenous) may vary between and within countries and regions.^45^^-^^47^

Given this ethnic heterogeneity, it is possible that equation performance may differ from one country to another. However, among the six studies that could be meta-analyzed in our study, five were performed in Brazil, where the ethnic composition differs from that of other countries in the region. As an example, while around 60% of the Brazilian population is Caucasian and less than 0.5% is Amerindian,^48^ in Peru around 60% of the population identifies themselves as Mestizo, 25% as Quechua or Aymara (Amerindians) and only around 6% as Caucasians.^49^ This prevents conclusions being drawn in relation to other Latin American countries where Amerindians represent an important proportion of the population. In this way, further studies comparing equations or trying to validate coefficients for other Latin American countries are needed.

### Implications

Our results suggest that in Latin American populations (mostly from Brazil), as in other populations, these equations do not vary greatly. However, CKD-EPI-Cr IDMS tends to have a non-significant better performance than MDRD-4 IDMS, in term of P30 and among people with GFR < 60 ml/minute/1.73 m^2^.

Nevertheless, it is necessary to highlight that the certainty of evidence was very low or low, which suggests that further well-designed studies are needed. In addition, extrapolation to other Latin American countries is difficult because almost all the meta-analyzed studies were performed in Brazil. Lastly, all the meta-analyzed studies used IDMS for creatinine calculation, which has to be taken into account in contexts that do not have IDMS.

### Limitations and strengths

Some limitations of this review should be considered: 1) not all studies had enough information to perform a meta-analysis on the outcomes of interest, even after the authors were consulted; and 2) we found differences in the characteristics of the populations included, but we were not able to perform any subgroup analysis to understand how these differences affected the accuracy of the formulas.^21^ The influence of other factors, such as the different causes of CKD or the medicines taken, was not studied either.^50^

In spite of these limitations, we believe that our study is important because this is the first systematic review that has compared the GFR equations in Latin American countries (mostly from Brazil), through a two-step sensitive search (the first in two international databases and one local database, and the second in the references and articles that cited each of the articles included in the first step). In addition, we performed a comprehensive search that including papers in Spanish and Portuguese, and the selection and extraction of data were performed in duplicate.

## CONCLUSION

We performed a systematic review to assess the performance of the CKD-EPI and the MDRD equations for estimating the GFR in Latin American countries. We found 12 studies and were able to meta-analyze six of them (five were conducted in Brazil). We found that the performances of CKD-EPI-Cr IDMS and MDRD-4 IDMS did not differ significantly, although CKD-EPI-Cr IDMS tended to have a non-significantly better performance in terms of P30 and among people with GFR ≥ 60 ml/min/1.73m^2^. However, since most of the meta-analyzed studies were from Brazil, the results cannot be extrapolated to other Latin American countries.
